# Supplementary material for: Establishment of conidial fusion in the asexual fungus Verticillium dahliae as a useful system for the study of non-sexual genetic interactions
Source: Curr Genet. 2021 Feb 13;67(3):471–85. doi: 10.1007/s00294-021-01157-4 (PMC8139932; doi:10.1007/s00294-021-01157-4)
Supplement: Supplementary file 1 — Supplementary file1 (DOCX 248 KB) [file 294_2021_1157_MOESM1_ESM.docx]

**Supplementary Figures**

ORIGINAL ARTICLE

**Establishment of conidial fusion in the asexual fungus *Verticillium dahliae* as a useful system for the study of non-sexual genetic interactions**

**Vasileios Vangalis^1^ • Michael Knop^2,3^ • Milton A. Typas^1^ • Ioannis A. Papaioannou^2,*^**

^1^ Department of Genetics & Biotechnology, Faculty of Biology, National and Kapodistrian University of Athens, Greece

^2^ Center for Molecular Biology of Heidelberg University (ZMBH), Heidelberg, Germany

^3^ German Cancer Research Center (DKFZ), DKFZ-ZMBH Alliance, Heidelberg, Germany

**^*^** To whom correspondence should be addressed: i.papaioannou@zmbh.uni-heidelberg.de

**
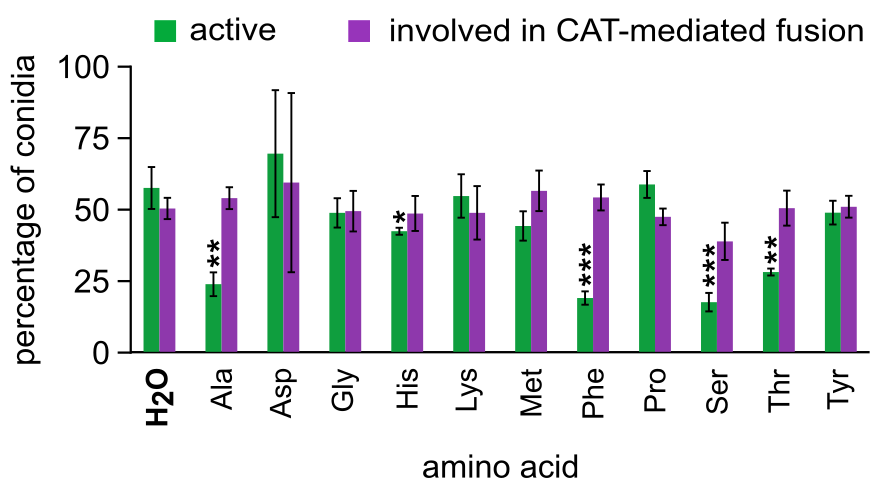
**

**Fig. S1** Influence of various amino acids on the frequencies of active conidia and active conidia involved in CAT-mediated fusion (*V. dahliae* isolate 123V). Each amino acid was tested in triplicate, and 300 conidia were analyzed per replicate. Bars = SD. Statistical significance of differences from the control was determined by Student’s *t*-test (* *p* ≤ 0.05, ** *p* ≤ 0.01, *** *p* ≤ 0.001). The results from the remaining amino acids are presented in Fig. 2a.

**
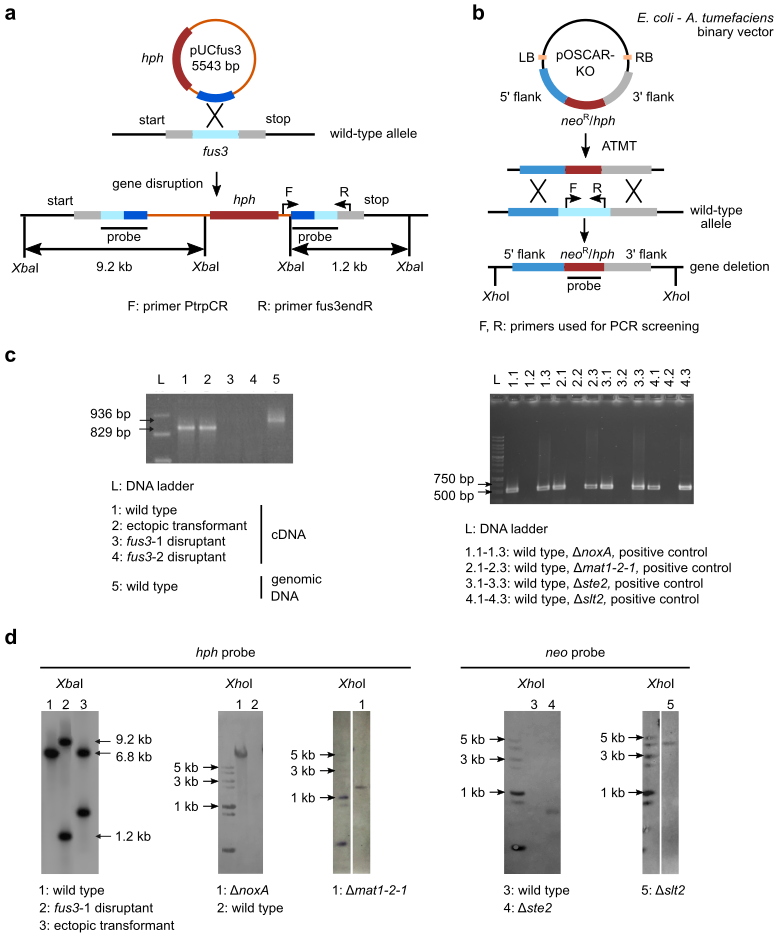
**

**Fig. S2** Construction and validation of null mutants used in this study. **a** Schematic representation of the gene disruption strategy that was followed for knocking out the *V. dahliae* homolog of *fus3*, using protoplast transformation of *V. dahliae* 123V. **b** A double homologous recombination-based strategy using *Agrobacterium tumefaciens*-mediated transformation was used for the deletion of the *V. dahliae* 123V homologs of genes *slt2*, *ste2*, *noxA* and *MAT1-2-1*. **c** PCR screening using gene-specific primers for disruption (*fus3*) or deletion (*slt2*, *ste2*, *noxA*, *MAT1-2-1*) mutants. **d** Validation of mutants by Southern hybridization experiments.
