# Supplementary material for: Establishment of conidial fusion in the asexual fungus Verticillium dahliae as a useful system for the study of non-sexual genetic interactions
Source: Curr Genet. 2021 Feb 13;67(3):471–85. doi: 10.1007/s00294-021-01157-4 (PMC8139932; doi:10.1007/s00294-021-01157-4)
Supplement: Supplementary file 2 — Supplementary file2 (DOCX 20 KB) [file 294_2021_1157_MOESM2_ESM.docx]

**Table S1** *Verticillium dahliae* strains used in this study

| **wild-type isolates** | | | | | | |
| --- | --- | --- | --- | --- | --- | --- |
| **strain** | | **VCG** | | **host** | | **origin** |
| T9 | | 1A | | cotton | | USA, CA |
| 123V | | 2A**^1^** | | tomato | | Greece |
| PH | | 2A | | pistachio | | USA, CA |
| Ls.17 | | 2B | | lettuce | | USA, CA |
| 115 | | 2B | | cotton | | Syria |
| Cf.38 | | 2B**^2^** | | chili pepper | | USA, CA |
| PCW | | 3 | | pepper | | USA, CA |
| BB | | 4A | | potato | | USA, ID |
| S39 | | 4B | | potato | | USA, OH |
| Ca.146 | | 6 | | bell pepper | | USA, CA |
| Ca.148 | | 6 | | bell pepper | | USA, CA |
| V13 | | HSI**^3^** | | cotton | | Spain |
| Cf.162 | | HSI**^2,3^** | | chili pepper | | USA, CA |
|  | |  | |  | |  |
| **^1^** The VCG of isolate 123V was determined in this study. | | | | | | |
| **^2^** Isolates Cf.38 and Cf.162 were originally misclassified in VCG 6. Their behavior was re-assessed and the VCGs provided here were determined by Papaioannou *et al*. (2014). | | | | | | |
| **^3^** HSI: Heterokaryon Self-Incompatible. | | | | | | |
|  | | | | | | |
| **constructed strains (this study)** | | | | | | |
| **strain** | **background** | | **genotype** | | **constructed with plasmid(s)** | |
| Ls.17-gfp | Ls.17 | | *sgfp, neo*^R^ | | pIGPAPA | |
| Ls-H1-mCherry | Ls.17 | | *nit1*, *VdH1-mCherry, ble*^R^ | | fusion PCR product | |
| Ls-H1-gfp | Ls.17 | | *nitM*, *NcH1-sgfp, hph* | | pMF357 | |
| Cf.38-H1-gfp | Cf.38 | | *nitM*, *NcH1-sgfp, hph* | | pMF357 | |
| 123-Δfus3 | 123V | | *fus3::hph* | | pUCfus3 | |
| 123-Δfus3-c | 123-Δfus3 | | *fus3::hph, fus3, neo*^R^ | | pUCfus3, *fus3*, pSD1 | |
| 123-ΔnoxA | 123V | | Δ*noxA::hph* | | pOSCAR-noxA | |
| 123-ΔnoxA-c | 123-ΔnoxA | | Δ*noxA::hph, noxA, neo*^R^ | | pOSCAR-noxA, *noxA*, pSD1 | |
| 123-Δmat1-2-1 | 123V | | Δ*mat1-2-1*::*hph* | | pOSCAR-mat | |
| 123-Δmat1-2-1-c | 123-Δmat1-2-1 | | Δ*mat1-2-1*::*hph, mat1-2-1,neo*^R^ | | pOSCAR-mat, *mat1-2-1*, pSD1 | |
| 123-Δslt2 | 123V | | Δ*slt2::neo*^R^ | | pOSCAR-slt2 | |
| 123-Δslt2-c | 123-Δslt2 | | Δ*slt2::neo, slt2, hph* | | pOSCAR-slt2, *slt2,* pUCATPH | |
| 123-Δste2 | 123V | | Δ*ste2*::*neo*^R^ | | pOSCAR-ste2 | |
| 123-Δste2-c | 123-Δste2 | | Δ*ste2*::*neo, ste2, neo*^R^ | | pOSCAR-ste2, *ste2*, pUCATPH | |
