# Supplementary material for: Establishment of conidial fusion in the asexual fungus Verticillium dahliae as a useful system for the study of non-sexual genetic interactions
Source: Curr Genet. 2021 Feb 13;67(3):471–85. doi: 10.1007/s00294-021-01157-4 (PMC8139932; doi:10.1007/s00294-021-01157-4)
Supplement: Supplementary file 3 — Supplementary file3 (DOCX 22 KB) [file 294_2021_1157_MOESM3_ESM.docx]

**Table S2** List of DNA oligonucleotides used in this study

| **oligo name** | **sequence (5’ to 3’)** | **template**  **(strain or plasmid)** | **construction/validation of strain or plasmid** |
| --- | --- | --- | --- |
| **nuclear labeling with H1-mCherry** | | | |
| HIST1-1 | GGAGGGTGGGCAGATGGAAAGAAGAGGGCG | Ls.17 | Ls-H1-mCherry |
| HIST1-2 | AGCAGCAGCCTTCTTCGGCGCGGCGGC |  |  |
| HIST1-3 | CGCTTCCGAGGGCCGAGGACAGCACGG |  |  |
| HIST1-4 | TGTGCCTGTGGGAGGAGCTCAAAGTGCCTC |  |  |
|  | | |  |
| HIST1-ADP | GCCGCCGCGCCGAAGAAGGCTGCTGCTGGAGCTGGTGCAGGCGCTGGAGCC | pAN8.1-mCherry |  |
| HIST1-PHL | CCGTGCTGTCCTCGGCCCTCGGAAGCGAAACAAGTGTACCTGTGCATTCTG |  |  |
|  | | |  |
| VdH1FusF | TCGACGAGCAGGTGGTGG | fusion PCR product |  |
| VdH1FusR | GCAAAGGGCAACGCACAAC |  |  |
|  | | | |
| **construction of knock-out strains** | | | |
| fus3F | GACGAYCAYTGCCARTAC | 123V,  123-Δfus3 | 123-Δfus3 |
| fus3R | GGAAYCTTCTTYTTGAA |  |  |
| fus3RTF | GCGGACCCTGCGTGAGATG |  |  |
| fus3endR | AGCGTGTCCTTGTGCTTGTC |  |  |
| PtrpCR | CTCCAGCCAAGCCCAAAAAG’3 |  |  |
|  | | | |
| fus-comp-F | CCCTACCTCCCTACGCTGCCTG | Ls.17 | 123-Δfus3-c |
| fus-comp-R | GAACCCGAACTGATATAAGTACC |  |  |
|  | | | |
| noxA-1B2r | GGGGACAGCTTTCTTGTACAAAGTGGAACTCAAACCCAGGCGCAAAACAACC | Ls.17 | pOSCAR-noxA |
| noxA-2B1r | GGGGACTGCTTTTTTGTACAAACTTGTATCCAACCAGGCCGTCCCAGTATC |  |  |
| noxA-3B4 | GGGGACAACTTTGTATAGAAAAGTTGTTGCGGGATGCCTTTTTGTGTCGTCT |  |  |
| noxA-4B3 | GGGGACAACTTTGTATAATAAAGTTGTAGGGTTTGGCGTTTCGTCTGGTTC |  |  |
|  | | | |
| NoxAintF | TGCCGGCAAGGACTACTG | 123V,  123-ΔnoxA | 123-ΔnoxA |
| NoxAintR | GGCGTGACACCGATACCA |  |  |
|  | | | |
| noxA-1B2r | GGGGACAGCTTTCTTGTACAAAGTGGAACTCAAACCCAGGCGCAAAACAACC | Ls.17 | 123-ΔnoxA-c |
| noxA-4B3 | GGGGACAACTTTGTATAATAAAGTTGTAGGGTTTGGCGTTTCGTCTGGTTC |  |  |
|  | | | |
| MAT1B2r | GGGGACAGCTTTCTTGTACAAAGTGGAACCTCGCCGTCGTGGAATGA | Ls.17 | pOSCAR-mat |
| MAT2B1r | GGGGACTGCTTTTTTGTACAAACTTGTGGGCGGGGCGAGTTTAGCA |  |  |
| MAT3B4 | GGGGACAACTTTGTATAGAAAAGTTGTTTCTTGCACAGGCTTGGATTAC |  |  |
| MAT4B3 | GGGGACAACTTTGTATAATAAAGTTGTAGGCTGCCCGGACTTTATG |  |  |
|  |  |  |  |
| VdMAT12F | CAGGCCCATGGTCGTGAT | 123V,  123-Δmat1-2-1 | 123-Δmat1-2-1 |
| VdMAT12R | CTAGCTGTGCTGCCACTTGTTC |  |  |
|  | | | |
| MAT1B2r | GGGGACAGCTTTCTTGTACAAAGTGGAACCTCGCCGTCGTGGAATGA | Ls.17 | pOSCAR-mat-c |
| MAT4B3 | GGGGACAACTTTGTATAATAAAGTTGTAGGCTGCCCGGACTTTATG |  |  |
|  | | | |
| 5flste2F | GAAACGACAATCTGATCCAAGCTCAAGCTATGCCATGCTCGCTCTCATAG | Ls.17 | pOSCAR-ste2 |
| 5flste2R | CAATATCAGTTAACGTCGCAGTGTGCTCTTCGTGAATG |  |  |
| 3flste2F | CACCAGCCCTGGGTTAGGCTCAGGCAAGGCAAC |  |  |
| 3flste2R | GCCTGCAGGTCGCGAGCGATCGCGGTACCGTTCACAGTGAGTGCTCG |  |  |
|  | | |  |
| ste2genF | CGAAGAGCACACTGCGACGTTAACTGATATTGAAGGAGCAC | pSD1 |  |
| ste2genR | GCCTTGCCTGAGCCTAACCCAGGGCTGGTGACGG |  |  |
|  | | | |
| Vdste2F | GCGCAGTAGGCCAGATCAGG | 123V,  123-Δste2 | 123-Δste2 |
| Vdste2R | GTCCCGTGCTCGTTTGGTG |  |  |
|  | | | |
| 5flste2F | GAAACGACAATCTGATCCAAGCTCAAGCTATGCCATGCTCGCTCTCATAG | Ls.17 | 123-Δste2-c |
| 3flste2R | GCCTGCAGGTCGCGAGCGATCGCGGTACCGTTCACAGTGAGTGCTCG |  |  |
|  | | | |
| 5flslt2F | GAAACGACAATCTGATCCAAGCTCAAGCTAGGTTTACCACGTCACAACTCG | Ls.17 | pOSCAR-slt2 |
| 5flslt2R | CAATATCAGTTAACGTCGATCTTGCGTCCCTGGAGG |  |  |
| 3flslt2F | CACCAGCCCTGGGTTAGGAGGATGGCAGGAGGAC |  |  |
| 3flslt2R | GCCTGCAGGTCGCGAGCGATCGCGGTACGCCCTACCAGATTCTCTAAGC |  |  |
| slt2genF | CAGGGACGCAAGATCGACGTTAACTGATATTGAAGGAGCAC | pSD1 |  |
| slt2genR | CTCCTGCCATCCTCCTAACCCAGGGCTGGTGACGG |  |  |
|  |  |  |  |
| Vdslt2F | CATTCCCCGCCCCGACAAC | 123V,  123-Δslt2 | 123-Δslt2 |
| Vdslt2R | GCAGCCCACGGACCAAACATC |  |  |
|  | | | |
| 5flslt2F | GAAACGACAATCTGATCCAAGCTCAAGCTAGGTTTACCACGTCACAACTCG | Ls.17 | 123-Δslt2-c |
| 3flslt2R | GCCTGCAGGTCGCGAGCGATCGCGGTACGCCCTACCAGATTCTCTAAGC |  |  |
