# Supplementary material for: Establishment of conidial fusion in the asexual fungus Verticillium dahliae as a useful system for the study of non-sexual genetic interactions
Source: Curr Genet. 2021 Feb 13;67(3):471–85. doi: 10.1007/s00294-021-01157-4 (PMC8139932; doi:10.1007/s00294-021-01157-4)
Supplement: Supplementary file 4 — Supplementary file4 (DOCX 22 KB) [file 294_2021_1157_MOESM4_ESM.docx]

**Table S3** Plasmids constructed and used in this study

| **plasmid** | **backbone** | **description** | **source** |
| --- | --- | --- | --- |
| pIGPAPA | - | P*_icl_*-*sgfp*-T_nosI_, *hph* | Horwitz *et al*., 1999 |
| pUCATPH | - | *hph* | Lu *et al*., 1994 |
| pSD1 | pBluescript II | P*_gpda_*, P*_trpc_*, *neo*^R^ | Nguyen *et al*., 2008 |
| pOSCAR | pPZP-RCS2 | *A. tumafaciens* binary vector | Paz *et al*., 2011 |
| pA-Hyg-OSCAR | - | *hph* | Paz *et al*., 2011 |
| pAN8.1-mCherry | pAN8.1 | GA-*mCherryFP, ble*^R^ | Ruiz-Roldan *et al*., 2010 |
| pMF357 | - | P*_ccg1_*-*NcH1*-*sgfp, hph* | Ishikawa *et al*., 2012 |
| pOSCAR-mat | pOSCAR | 5’H*_Vdmat_*-*hph*-3’H*_Vdmat_*  (H: 2.0 kb-long homology arms) | This study |
| pOSCAR-noxA | pOSCAR | 5’H*_VdnoxA_*-*hph*-3’H*_VdnoxA_*  (H: 2.0 kb-long homology arms) | This study |
| pOSCAR-slt2 | pOSCAR | 5’H*_Vdslt2_*-*neo*^R^-3’H*_Vdslt2_*  (H: 2.0 kb-long homology arms) | This study |
| pOSCAR-ste2 | pOSCAR | 5’H*_Vdste2_*-*neo*^R^-3’H*_Vdste2_*  (H: 2.0 kb-long homology arms) | This study |
| pUCfus3 | pUCATPH | *fus3*, *hph* | This study |
